# Low-dose ketamine infusions for chronic pain management: Does this qualify as evidence-based practice?

**DOI:** 10.1177/20494637231182804

**Published:** 2023-06-21

**Authors:** Harry M Griffiths

**Affiliations:** Peninsula Schools of Medicine, 12208 University of Plymouth, Plymouth, UK

**Keywords:** Pain, chronic pain, pain management, pain postoperative, pain clinics

## Abstract

Chronic pain is becoming increasingly prevalent and burdensome both worldwide and in the United Kingdom. Due to the complexity of chronic pain and the therapeutic challenge associated, management is often difficult and requires multidisciplinary care encompassing a combination of pharmacological and non-pharmacological strategies. Conventional analgesic treatments, such as opioids and anticonvulsants, are effective in less than half of chronic pain sufferers and are typically limited to short-term use to prevent complications associated with long-term use such as tolerance and dependence. Consequently, research and clinical interest in alternative management options for chronic pain have increased in recent years, with ketamine being one example under investigation. However, since ketamine has been licensed as an anaesthetic for decades, it has bypassed the traditional scrutinous drug development sequence that is typically seen for therapeutics marketed for pain. As such, data supporting the unlicensed administration of ketamine for chronic pain management is lacking and is being outpaced by the rates of off-label use in pain clinics. Recent limited evidence suggests that ketamine, when given as an intravenous infusion in subanaesthetic doses for refractory pain patients, may provide modest analgesic effects in nearly all aetiologies of chronic pain, with side effects common but typically mild. However, there are concerns over the safety of this practice due to the paucity of robust supportive evidence and the accompanying lack of clinical guidelines or standardised protocols. This review shall summarise the literature examining the use of subanaesthetic-dose ketamine infusions for chronic pain to comment on the current level of evidence, with limitations of existing research and future recommendations discussed.

## Introduction

Chronic pain is exceptionally prevalent and continues to result in significant health, societal, and economic burden worldwide.^1^ Due to the complexity of chronic pain perception, to which psychological and social factors contribute, management is often challenging and requires multimodal strategies.^2^ Across all aetiologies, it is estimated that conventional analgesic treatments, such as opioids, antidepressants, and antiepileptics, are effective in just 30–40% of chronic pain patients, with the rest suffering inadequate pain control.^3,4^ Moreover, these drugs are limited by complications preventing long-term use as evidenced by the ongoing opioid epidemic in the United States (US), where mortality is fuelled by immoderate opioid prescribing^5^; a public health crisis that, according to recent evidence, the United Kingdom (UK) is at risk of mirroring.^6^ Consequently, the need for safe and effective pharmacological alternatives to complement the already-complex chronic pain management arsenal, particularly for refractory cases, is increasingly evident.^7^ An emerging candidate is ketamine, an N-methyl-D-aspartate (NMDA) receptor antagonist, which is gaining traction in the field of chronic pain.^2,8^ While ketamine has been used medically as an anaesthetic since the 1970s,^9^ with some efficacy in the psychiatric literature,^10^ there has been a recent surge in clinical and research interest for its role in chronic pain management in subanaesthetic intravenous (IV) doses, along with other formulations such as intranasal S-ketamine (esketamine).^11–13^ However, off-label ketamine prescribing rates for chronic pain are outpacing the supporting clinical evidence,^14^ suggesting a potential widespread failure in evidence-based prescribing and hence attracting the ‘Wild West’ phrasing in both the academic and lay press.^1,11,15,16^

## Literature review

### Background

Historically, the phenomenon of pain, including chronic, was thought to have a linear, causal relationship between tissue injury and the resulting pain perception from patients.^17^ It was long assumed that upon resolution of the underlying lesion, the pain would naturally cease thereafter, without interference.^18^ However, owing to the increasing prevalence of chronic pain and the improvement in medical standards resulting in longer life expectancies and thus greater suffering potential,^19^ it is well-established that chronic pain perception is characterised by a complex amalgamation of physical and biopsychosocial factors that are unique to each sufferer.^20,21^ These contributing factors vary significantly in their influence and immitigability, from genetics to previous pain experiences, to concurrent mental health burdens and the social pressures of finances and relationships.^21^ Moreover, the complexity of chronic pain extends to the associated therapeutic challenge, which typically requires multidisciplinary input and remains a distressing issue due to its exceptional prevalence.^22^ Staggeringly, a systematic review of 139,933 adult residents from seven epidemiological studies in the UK concluded a pooled chronic pain prevalence rate of 43.5% (95% confidence interval (CI), 38.4–48.6%), of which 10.4–14.3% were defined as moderate-to-severely disabling.^23^ This translates to a significant public health concern given the threat to relationships, well-being, and work that chronic pain typically imposes.^21^ In fact, chronic pain is associated with higher divorce and suicide rates,^10,24^ in addition to a reduced life expectancy when accounting for other variables.^25^

### The aetiology of chronic pain

When pain is associated with evident tissue damage or an underlying disease process, and is transient (from days to weeks), it is referred to as acute pain.^26^ Although there is no strict threshold, the International Association for the Study of Pain (IASP) defines chronic pain as exceeding 3 months, whether it be accompanying a chronic disease process such as fibromyalgia or persisting after healing from trauma such as chronic postsurgical pain.^27^ Chronic pain may be aetiologically categorised via mechanism into neuropathic, nociceptive, and nociplastic, and via location into central, peripheral or mixed.^1,21^ Understanding the aetiology of chronic pain helps to inform the appropriate analgesic management. For example, in the literature, neuropathic pain is favourably treated with antidepressants, gabapentinoids and ketamine; meanwhile, nonsteroidal anti-inflammatory drugs (NSAIDs) are generally considered ineffective here but are superior for nociceptive pain.^1^ However, these theories are largely drawn from studies of animal models, and in reality, a single therapeutic agent may provide efficacy across several aetiological categories of chronic pain.^11^ This may be explained partly by the overlap of pain phenotypes expressed by some disease processes, in addition to the variability in patient pain perception which thus translates to the variability in analgesic response.^1,2^ As such, clinical trials have demonstrated efficacy of therapeutics for other pain aetiologies from which they were intended, including NSAIDs for neuropathic pain and gabapentinoids for nociceptive pain.^28–30^ Hence, due to the heterogeneity of many pain-inducing syndromes, many researchers consider the classifications of pain a continuum rather than distinct identities, and this is generally accounted for in modern multimodal analgesic strategies.^1,2,21^

The IASP defines neuropathic pain as that resulting from disease or damage to the somatosensory nervous system^27^; it is typically described as a shooting or shocking sensation and may be associated with sensory abnormalities such as numbness and allodynia (a painful response to non-painful stimuli).^31^ Examples of causes of central neuropathic pain include (but are not limited to) traumatic (spinal injury), autoimmune (multiple sclerosis), and inflammatory (transverse myelitis). Peripherally, examples of causes include any type of peripheral neuropathy such as infective (herpes zoster), nerve damage (carpal tunnel syndrome), or ischaemic (peripheral vascular disease and diabetic neuropathy).^21^ Chronic neuropathic pain syndromes that have been studied with subanaesthetic-dose ketamine infusions (SDKIs) include phantom limb pain, postherpetic neuralgia, spinal cord injury, and oncological neuropathic pain.^14,31,32^

In comparison, nociceptive pain, the most common type of chronic pain, results from neural pathway stimulation secondary to either physical stimuli or a disease process affecting somatic structures, such as arthritis and most spinal pain syndromes.^21^ In other words, nociceptive pain is caused by tissue damage or the potential of damage stimulated by a disease process, and may be described as throbbing, aching, or a pressure sensation.^21^ Peripheral nociceptive pain may arise from somatic organs such as joints, muscles and skin, while central nociceptive pain may occur from visceral organs such as mucosal injury from peptic ulcer disease, ischaemia of the bowel, or luminal obstruction of the biliary or urogenital systems.^21^ Finally, nociplastic pain occurs due to altered nociceptive pathways despite no evidence of physical injury or inflammation to peripheral nociceptors; examples that have been studied with SDKIs include complex regional pain syndrome (CRPS), headaches, and fibromyalgia.^11,33^ As such, in contrast to neuropathic and nociceptive pain, the pattern of nociplastic plain is typically diffuse and not confined to one anatomical region.^21^

Interestingly, cancer pain may encompass all three types of chronic pain and has also been studied with SDKIs.^34^ However, due to the significant overlap of pain phenotypes associated with cancer, it is currently unclear whether cancer pain should be treated the same as noncancer pain.^21^ While the Centre for Disease Control (CDC) guidelines on opioid use recommend that cancer pain should be identified and managed separately from noncancer pain,^35^ the US Food and Drug Administration (FDA) assert that the mechanisms between the two are identical and hence should be treated equally.^36^

### The introduction of ketamine

Ketamine was first synthesised in 1962 as an alternative anaesthetic agent to phencyclidine,^7^ from which it is derived, in an attempt to find an agent with a faster onset and improved safety.^2,15^ Although used less favourably for induction of anaesthesia today, it remains clinically advantageous in humans for perioperative haemodynamic control and postoperative analgesia.^15^ However, in recent years, poorly regulated ‘ketamine clinics’ for chronic pain (and depression) have sprouted worldwide,^11,15^ with frequent administration reported in the US, the Netherlands, France, South Korea, and the UK.^8,37–39^ As such, ketamine for chronic pain has bypassed the traditional, rigorous drug development sequence involving initial animal testing, then human experimental studies and clinical trials assessing efficacy and safety, followed by strict surveillance.^11^ The first ketamine clinic that opened in the UK was in Bristol, charging £6000 for a course of low-dose infusions with psychotherapy for depression.^16^ Since ketamine is licensed as an anaesthetic in the UK, off-label use for chronic pain is not illegal.^8^ However, administration in the National Health Service has yet to receive approval from the National Institute for Health and Care Excellence as a cost-effective treatment, with no signs of imminency.^16^ Hence, there are concerns that prescribing SDKIs may be unethical if motivated by profit above providing high-quality evidence-based care where the mechanisms of treatment are well-understood.^2^ This has led to a rise in observational and experimental studies examining the use of SDKIs in chronic pain of many aetiologies,^3,9,11^ in addition to expert consensus guidelines published by a collaboration of several American pain bodies for this specific indication.^1^

### How does ketamine exert its effects to aid in chronic pain management?

NMDA receptor activation by glutamate, the primary excitatory neurotransmitter in the central nervous system (CNS), is a principal mechanism behind cognition, mood regulation, opioid tolerance, and chronic pain.^1,8^ Through both animal and human studies, it is known that NMDA receptors play a key role in the transition from acute to chronic pain through central sensitisation and the ‘wind-up’ mechanism, whereby activation thresholds of pain are reduced over time.^1,12,37^ Hence, ketamine, an uncompetitive NMDA receptor antagonist,^9^ is thought to exert its primary analgesic effect through the reversal of these mechanisms,^3,11^ particularly for neuropathic pain, where most SDKI research exists as driven by this theory.^14,32^ However, ketamine has an affinity to an impressive list of receptors, including opioid (mu, kappa and sigma), dopaminergic, serotonergic, monoaminergic, anticholinergic, muscarinic, gamma-amino-butyric acid (GABA), and many more, permitting its antidepressive, analgesic, and psychomimetic effects.^1,2,8^ As a result, the efficacy of ketamine has been demonstrated in nociceptive and nociplastic pain syndromes,^1,14^ thus making NMDA receptor blockade an oversimplification of the analgesic mechanism of ketamine.^2^ Further, several clinical trials have supported the role of ketamine in treatment-resistant depression and alcohol and heroin withdrawal, which may be explained by its wide receptor affinity extending to the dopaminergic, serotonergic, and GABA receptors.^3,40–42^ Esketamine, the S (+)-enantiomer of ketamine, possesses approximately a four-fold affinity to NMDA receptors and a two-to-three-fold affinity to opioid receptors with a shorter duration of action compared to the traditional racemic form,^1,3^ thus making it an attractive candidate for recent research into pain and depression therapy.^37,43,44^ At present, the specific mechanistic details underpinning the role of ketamine in chronic pain are poorly understood but are inspiring new theories and uses of ketamine; for example, the opioid-receptor affinity has driven research into the use of ketamine as an opioid-sparing analgesic adjunct for cancer pain, with promising but limited results.^34,45^

Reported protocols vary significantly,^3^ but a typical SDKI is less than 0.5–1 mg/kg, or a dose that will cause minimally acceptable levels of sedation.^2,14^ This may be considered a low dose given that 1–4.5 mg/kg is required for anaesthesia induction and 1–6 mg/kg/hr is needed for anaesthesia maintenance.^1^ An SDKI of 0.5 mg/kg will result in a plasma concentration of 70–200 ng/mL, below the threshold to achieve a loss of consciousness (>500 ng/mL) or deep anaesthesia (2000–3000 ng/mL).^2^ In a recent meta-analysis, the median infusion dose was found to be 0.35 mg/kg for a duration of 5 h.^11^ The most common form of administration of ketamine is the IV route, which has the highest bioavailability compared with, in descending order, intramuscular (∼93%), intranasal (∼45%), sublingual (24–30%), and oral (17–24%).^2^ Hence, IV administration has been the most studied route in chronic pain as other formulations have highly variable pharmacokinetics and durations of action, in addition to inferior monitoring resources.^1,11,14^ Notwithstanding, the prospect of improved convenience and safety offered by other formulations, such as intranasal, is driving research into the clinical utility of ketamine further, with promising results from preclinical studies of both postoperative pain and depression.^44^ In a recent randomised double-blind study, intranasal esketamine combined with antidepressant therapy had modest efficacy over an antidepressant with intranasal placebo in reducing symptoms of major depressive disorder over 4 weeks.^13^ Although the IV route is the most invasive, SDKIs are typically reserved for refractory chronic pain patients; therefore, the benefits may outweigh the risks in most situations.^37^ Further research examining the use of non-intravenous formulations of ketamine and esketamine for chronic pain, including oral, intranasal and iontophoretic, may eventually reduce the frequency of SDKIs needed for adequate pain control to achieve a more favourable safety profile.^7^

Approximately 80% of ketamine is metabolised in the liver to norketamine, which has roughly a third of the antinociceptive affinity of ketamine but a longer plasma half-life.^2^ Interestingly, the effects of IV ketamine on chronic pain, which can last several months,^4^ have been reported far beyond the elimination half-life of 6 h; this has two possible explanations.^1,2^ First, the longer half-life of norketamine could explain the downstream effects of prolonged SDKIs.^2^ Second, evidence from animal studies suggests that ketamine affects the affective-motivational dimension of chronic pain; that is, beyond the sensory-discriminative element (peripheral nerve stimulation), and higher up the CNS where emotional arousal and experience of pain are processed.^1,2^ This could explain why relief from chronic pain and depression has been reported up to 12 weeks after a single infusion,^11,32^ significantly outlasting the physical sensory changes caused by ketamine.^2^ This modulation of the affective-motivational component of pain perception may also explain the reported efficacy of ketamine infusions for treatment-resistant depression and posttraumatic stress disorder.^2^

### What is the current evidence behind SDKIs for chronic pain?

Recent systematic reviews, meta-analyses, and primary studies have reported modest improvements in chronic pain outcomes from SDKIs.^4,7,11,12,31–33,46^ Although there is no formal consensus, a significant effect that may translate to clinical utility is typically determined by most researchers as a ≥2 reduction in pain scores (1–10), or a ≥30% reduction in pain intensity as suggested by US guidelines.^37^ A meta-analysis of randomised controlled trials (RCTs) by Michelet and colleagues (2017) concluded that a single dose of ketamine was effective in reducing chronic noncancer pain at 1, 2, 8, and 12 weeks but not 4 weeks; however, they included non-IV formulations which may have skewed their results.^7,11^ Similarly, Orhurhu *et al.* (2019) reported significant reductions in pain scores from one SDKI at 48 h, 2 weeks, and 8 weeks but not 4 weeks; meta-analysis of these data resulted in a mean difference (MD) reduction in pain scores of −1.83 (95% CI, −2.35 to −1.31).^11^ Further, high-dose SDKIs demonstrated higher efficacy than low-dose compared with placebo (high-dose MD, −2.11; 95% CI, −2.87 to −1.35; low-dose MD, −1.30; 95% CI, −2.01 to −0.59).^11^ However, SDKIs resulted in a significantly higher risk of adverse events compared with placebo, including nausea (relative risk (RR), 3.52; 95% CI, 1.74–7.14) and psychomimetic symptoms (RR, 5.92; 95% CI, 2.95–11.89).^11^ Interestingly, no significant differences in pain reduction were observed between patients with neuropathic compared with nociceptive and nociplastic pain syndromes.^11^

These data suggest that SDKIs offer a promising treatment option for chronic pain aetiologies of both neuropathic and nociceptive origin that are refractory to conventional treatments but may be limited by side effects, which are common but overall mild and have pharmacological management options.^1^ For example, clonidine and midazolam have been shown to reduce the psychomimetic effects of ketamine when administered as an adjunct to infusion therapy, which may become standard inclusions as the evidence base strengthens.^2,14^ In a systematic review of 2893 patients from 53 trials, the number needed to harm for hallucinations induced by ketamine was 35, reducing to 21 with benzodiazepine premedication.^47^ While short-term ketamine use in the clinical setting has no known prolonged psychological effects lasting beyond 24 h, the complications associated with repeated or long-term use when prescribed are poorly understood and thus require caution.^8^

Serious hepatic, cardiac, and urogenital adverse events are mostly found in chronic, recreational users,^2^ with just one report of ulcerative cystitis in the medical setting.^48^ For example, elevated liver enzymes have been reported from SDKIs but are transient and resolve in convalescence.^2,32^ Elevated cardiovascular signs have also been reported from SDKIs due to their negative inotropic effect which may require additional monitoring but may be managed with clonidine or beta-adrenoreceptor blockers, although the evidence supporting this is limited.^1,2,49^ While these serious adverse events appear to be dose-dependent, the psychomimetic and nauseating effects have been reported in nearly all doses.^2^ Therefore, evidence suggests that SDKIs are associated with transient but reversible psychomimetic effects with no dose-dependent relationship within the subanaesthetic range.^2,11^ However, as ketamine is excreted in the liver and kidneys, hepatic or renal insufficiency may predispose patients to dose-dependent side effects concerning the cardiovascular, hepatic, and urogenital systems.^2^ Nevertheless, psychotic disorders, cardiovascular disease, and hepatic and renal disease remain relative contraindications to SDKIs until more data can elucidate a safe protocol in these populations where the benefits outweigh the risks.^1^ Currently, active psychosis, substance abuse, and delirium are considered absolute contraindications to SDKIs.^2^ Given that most existing literature exploring the safety of ketamine focuses on the anaesthetic dose range or recreational abusers, concluding the risks of recurrent ketamine infusions in the subanaesthetic dose range remains challenging at present; however, severe hepatotoxic or urogenital side effects appear exclusively reserved for chronic abusers of ketamine.^1,2^

The benefit of a single high-dose SDKI over a low-dose regimen was also demonstrated in a 1-year follow-up study of 256 chronic pain patients by Corriger *et al.* (2021), who suggested that an infusion of 0.5 mg/kg might be too low to produce a lasting analgesic effect.^4^ In this study, the first to follow-up beyond 3 months, reduction in pain intensity persisted at 12 months post-infusion from baseline (baseline mean pain score, 6.8 ± 1.8; at 12 months, 5.7 ± 1.8).^4^ However, side effects were common after 1 week (108/218, 50%), including disorientation, fatigue, nausea, and headache, but this decreased to 28/172 (16%) after 1 month and continued to decrease over time.^4^ Importantly, it is possible that these data are confounded by changes in disease course or additional pharmacological management; this also applies to other studies, and the magnitude of this confounding effect is unclear.^9^ Notwithstanding, this report is just one example of the high drop-out rates seen in primary studies examining SDKIs in chronic pain; hence, most original studies are small RCTs with methodological limitations which reduces the robustness of subsequent meta-analyses.^1,2,32^

In addition to the limited power from small sample sizes, significant study heterogeneity concerning study populations and infusion protocols, along with short and varying follow-up durations, are major sources of bias that complicate conclusions on the effectiveness of SDKIs.^2,11^ Moreover, blinding has been shown to be challenging in some RCTs due to the experience or anticipation of psychomimetic effects,^1^ with one trial reporting that 75% of participants guessed their treatment correctly,^50^ and 93% in another small trial of 30 participants.^51^ Systematic reviews have concluded that the effect size of a treatment may be increased by 35% when blinding is absent and by 13–25% when blinding is unclear, which may exaggerate the promising results seen in recent RCTs.^1^ An overview of the current evidence of the use of SDKIs for chronic pain is summarised in Table 1.

**Table 1.** Summary of recent studies evaluating SDKIs for chronic pain.

Study	Study design	Follow-up duration	Patient selection	Treatment group	Control group	Outcome measure(s)	Analgesic outcomes
Corriger et al.^4^	Prospective multicentre observational study, outpatient	12 months	*N* = 256, age ≥18 years with chronic pain ≥6 months	Single SDKI, dose and duration variable	N/A	Numeric pain scale (0–10) every month	Statistically significant reduction in pain intensity at 1 month, 6 months, and 1 year; effect size at 1 year 0.61 (95% CI, 0.40–0.80; *p* < 0.001)
Amr^62^	Double-blind placebo-controlled trial, inpatient	3 weeks	*N* = 40, severe neuropathic pain ≥6 months	SDKI (5 h daily for 7 days at 0.22 mg/kg/h) with gabapentin 300 mg three times daily	Normal saline with gabapentin 300 mg three times daily	Visual analogue pain score (0–10) at baseline, week 1 and week 3	Significant reduction in pain in ketamine group at weeks 1 and 2 (largest effect at week 1), not significant by week 3
Schwartzman et al.^63^	Double-blind placebo-controlled trial, outpatient	12 weeks	*N* = 19, complex regional pain syndrome types I and II	SDKI (4 h daily for 10 days at 0.35 mg/kg/h) with midazolam and clonidine	Normal saline with midazolam and clonidine	Numeric pain score (0–10), McGill pain questionnaire, quantitative sensory testing, motor function, quality of life: at weeks 2, 4, 8 and 12	Significant reduction in pain intensity in ketamine group throughout 12 weeks for secondary outcomes but not primary outcome
Noppers et al.^50^	Double-blind placebo-controlled trial, outpatient	8 weeks	*N* = 24, fibromyalgia	Single SDKI (0.5 mg/kg for 30 min) with esketamine	Midazolam 5 mg	Visual analogue pain score (0–10), fibromyalgia impact questionnaire, quantitative sensory testing: Baseline, week 1 and week 8	Pain scores significantly lower in ketamine group 15 min post-infusion, but not significantly different beyond 15 min
Mitchell and Fallon^64^	Double-blind placebo-controlled trial, inpatient	5 days	*N* = 35, ischaemic limb pain	Single SDKI (0.6 mg/kg for 4 h) with opioids	Normal saline with opioids	Numeric pain scale (0–10) at baseline, 24 h and 5 d after infusion	Significant reduction in pain and improvement in activity level in ketamine group, maximal benefit at 5 d
Sigtermans et al.^51^	Double-blind placebo-controlled trial, inpatient	12 weeks	*N* = 60, complex regional pain syndrome type I	S-ketamine infusion titrated to patient benefit (range 0.07–0.43 mg/kg/h)	Normal saline	Numeric pain scale (0–10) each week of study	Pain scores significantly lower in ketamine group for weeks 1–8, no longer significant by week 12

SDKI: subanaesthetic dose ketamine infusion; h: hours; d: days; mg: milligrammes; kg: kilogrammes; *N*: number of participants; CI: confidence interval.

It has been suggested that esketamine may have superior efficacy and reduced psychomimetic complications compared with traditional racemic ketamine in studies examining its role in the management of pain and depression.^52^ Intranasal esketamine was recently approved by the US FDA for treatment-resistant depression (Spravato; Johnson & Johnson),^53^ which may aid future research into its utility in pain of varying aetiologies (acute, chronic, cancer-related, postoperative, and opioid-refractory). While clinical trials examining the efficacy of esketamine for chronic pain are currently ongoing (NCT04938713, NCT05160493, NCT04847245, NCT04666623), primitive studies have reported modest improvements in outcomes relating to postoperative pain and treatment-resistant depression.^54–57^

The significant variability in existing protocols and the lack of direct comparisons prohibit the conclusion of an optimal dose from the literature, particularly for vulnerable populations, which remains a vital area of research needed to increase the safety of SDKIs and improve the robustness of guidelines.^11,14,37^ Surveys of pain clinics in France, South Korea, and the Netherlands have demonstrated this variability between practices, including the dose, duration, and monitoring facilities, but they report that serious adverse events are rare.^37–39^ Therefore, although these widely varied protocols suggest a lack of evidence-based prescribing, they appear to be well-tolerated in general, particularly when accounting for the likely significant disease burden of eligible patients prior to SDKI administration.^4^ In addition, the monitoring and documentation of side effects in both the research and clinical setting may be aided by the recently developed Ketamine Side Effect Tool (KSET).^58^ Some authors have suggested that a low-dose infusion over a prolonged period may be superior to a high-dose infusion over a shorter period to achieve a similar pain reduction with a lower risk of side effects, but this comparison remains understudied.^2,14^ Further, this may have unfavourable practical implications concerning monitoring costs and treatment burden for patients.^14^

### How can the evidence base and the safety of SDKIs be improved?

While preclinical studies of ketamine use in animal models and some experimental trials in humans for acute and chronic pain have shown promising results, clinical trials with long-term follow-up are needed to determine whether these benefits are safely reproducible in humans.^2^ Most existing studies examining SDKIs for chronic pain are small uncontrolled trials with either absent or suboptimal blinding; efforts to increase sample sizes, reduce dropout rates, and improve blinding effectiveness must be addressed prior to future syntheses of evidence.^1,7,12^ In addition, the heterogeneity in study design and patient population in existing SDKI research should be acknowledged before drawing conclusions on its effectiveness in chronic pain.^1,7^ In the absence of robust guidelines, data on optimal patient selection, adverse event mitigation, pre-infusion risk assessment, and the use of adjuvant medications are lacking.^2,11,14,59^ With these limitations taken into consideration, it is evident that funding for future research including large multicentre RCTs is needed to address the gaps in the evidence base and to refine current treatment delivery protocols for various patient populations.^11,32,59^ Moreover, since the goal of chronic pain management is to reduce pain to an acceptable level to promote functional recovery,^2,26^ it is unclear how the observed improvement in pain scores from SDKIs translates clinically to an improvement in functional capacity.^32^ Identifying further outcome measures beyond numerical pain scales should be considered to help further elucidate the risk-benefit profile of SDKIs, such as longitudinal measurements of functional capacity and independence.^12^

Regarding treatment duration, the consequences of prolonged clinical (non-recreational) administration are not sufficiently evidenced to make recommendations as to when a refractory chronic pain patient can safely terminate treatment with SDKIs while avoiding long-term complications or dependence.^1^ As such, there is no clear exit strategy for these patients where satisfactory pain relief can be provided with minimal side effects; longitudinal studies are needed to address this concern with SDKI treatment.^2^ Further, there is scope for determining the role of oral and intranasal ketamine formulations in tailoring SDKI treatment and aiding in its cessation.^9^ In addition, the risk-benefit ratio of SDKIs may be tipped favourably towards greater benefit if individuals likely to respond to treatment are identified prior to recruitment based on their pain phenotype.^4^ The most common causes of chronic pain, such as back pain and musculoskeletal disorders, should be prioritised for investigation to determine the effectiveness of SDKIs in these predominantly nociceptive and nociplastic pain syndromes.^1^

Another major source of bias in existing studies includes the confounding effect from the numerous analgesic medications eligible patients are usually taking when initiating SDKI therapy.^4,9,12^ Indeed, in practice, SDKIs may be offered as part of a multidisciplinary treatment approach rather than as monotherapy, since it is recommended as a third-line option in refractory chronic pain patients.^1^ However, for research purposes, these confounding variables should be controlled in future trials to determine the efficacy and safety of SDKIs in isolation and thus aid future recommendations.^4,9^ Unfortunately, since ketamine is labelled as an experimental or investigational drug in chronic pain, in addition to the lack of industry funding for generic formulations, it is unclear whether future large, robust studies will be performed to catch up with the rates of administration of SDKIs and ensure that this area of prescribing is evidence-based.^2,11^ Owing to its recent approval from the FDA, research into intranasal esketamine is gaining momentum which may facilitate subsequent research grants into IV ketamine and esketamine for pain management in the future. However, at present, the clinical utility of intranasal esketamine for chronic pain is unknown.^2^

Just one guideline has been published describing the use of SDKIs in chronic pain, which is an expert consensus statement due to the limited supportive evidence.^1^ As such, many recommendations are indistinguishable between expert opinion and expert evidence and thus require cautious interpretation,^60^ and policies may not resonate with prescribers unless they are data-driven.^61^ While medical practitioners have an obligation to prescribe high-risk medications judiciously and in moderation, the health and societal burden imposed by chronic pain and the limited effectiveness of current therapeutics may warrant trialling alternatives such as SDKIs if compromising patient safety can be avoided.^11,15^ To facilitate this, identification of patients likely to be at high risk of complications from SDKIs, using informed selection criteria from existing research, can facilitate the allocation of additional resources for monitoring and adjuvant medications to mitigate adverse events if necessary.^1^ In addition, establishing registries of ketamine clinics, where data are stored and analysed, can help clarify the risk of SDKIs to the wider population and reduce the reliance on data from small RCTs.^1^ This could also elucidate the optimal dose, infusion rate, and infusion duration for chronic pain patients of various disease aetiologies and sociodemographic factors.^15^ Moreover, since chronic pain management is typically more effective when multidisciplinary,^15,21^ the impact of SDKIs in various combinations with other pharmacological and non-pharmacological treatments requires further examination.^15^

## Conclusion

With the burden of chronic pain accumulating, and in light of the risks associated with long-term opioid use, the need for safe and effective alternatives is increasingly evident. SDKIs offer a promising alternative for refractory chronic pain patients who are resistant to conventional treatments. Modest reductions in pain scores have been observed across a wide range of types of chronic pain. While side effects of SDKIs are common but typically mild, serious adverse events are dose-dependent and are hence usually rare when doses are limited to the subanaesthetic range. However, in the absence of large, robust studies, the evidence supporting these outcomes for SDKIs is limited. In contrast to conventional therapeutics, determining whether there are enough data to suggest that SDKIs for chronic pain are sufficiently evidence-based remains unclear. Clarity regarding the indications and contraindications of SDKI therapy for various patient populations should improve as new evidence emerges. Further well-conducted research into SDKIs comparing treatment protocols, adjuvant medications, and different patient populations is warranted so that this area of practice can be more evidence-based.

## Appendix

### Abbreviations

CDC: Centre for Disease Control
CI: Confidence interval
CNS: Central nervous system
CRPS: Chronic regional pain syndrome
FDA: Food and Drug Administration
GABA: Gamma-amino-butyric acid
IASP: International Association for the Study of Pain
IV: Intravenous
KSET: Ketamine Side Effect Tool
MD: Mean difference
NMDA: N-methyl-D-aspartate
NSAID: Non-steroidal anti-inflammatory drug
SDKI: Subanaesthetic-dose ketamine infusion
RR: Relative risk
RCT: Randomised controlled trial
UK: United Kingdom
US: United States
